# Progress in Anæsthetics

**Published:** 1900-07-14

**Authors:** 


### PROGRESS IN ANÆSTHETICS.

**Preparation of the Patient.—Choice of an Anæsthetic.**—The necessity for careful preparation of the patient by abstinence from food, and the administration of purgatives, is now generally recognised by anæsthesists. Temple[1] suggests that in emergency operations the stomach be washed out by means of a syphon tube, thus diminishing the risks from vomiting. More attention should be paid to clothing patients warmly, and keeping them covered during the operation. When there is profound shock, brandy and beef-tea or normal saline fluid should be given per rectum before anæsthetisation, or digitalin and strychnine administered hypodermically. In neurasthenic, hysterical, and excitable patients, morphia given before the operation is often attended with the best results. Spraying the nose with cocaine diminishes the sense of suffocation and the unpleasant odour sometimes experienced with ether, but from its depressent effects upon the heart this procedure cannot be indulged in with impunity. With regard to the choice of an anæsthetic: up to five years of age, A.C.E. is the best; after that age ether. In chest diseases use chloroform or A.C.E.; in renal cases, chloroform. In a discussion on anæsthetics at the New York County Medical Association, Dr. Wyeth[2] maintained that chloroform was a more dangerous anæsthetic for children than ether. This idea was strongly combated by Hare, who pointed out that ether was much more likely to set up inflammation of the respiratory tract, and that the fresh heart of the child was better able to stand chloroform than that of the adult. Several speakers remarked on the value of hypodermic injections of atropine before anæsthetisation. In feeble persons, bandaging the limbs and the application of an abdominal compress lessened the risk. The tone of this meeting indicated a reaction in favour of chloroform as against ether for a general anæsthetic.

**Nitrous Oxide.**—The sense of suffocation and oppression experienced in taking gas may be entirely obviated by using a wide tube, free valves, and sufficient gas pressure. Flux[3] uses a face piece open at the top, and fitting closely to the face below. Into this space gas falls from the end of a supply pipe by its own weight, and is mixed with a small quantity of air. Anæsthesia is induced, and may be kept up indefinitely. Seven to eight gallons, as a rule, suffices for a tooth extraction. Gas[4] is contra-indicated in very obese patients, in cases with arterial degeneration, or right heart obstruction. It may be used for prolonged operations, provided that muscular relaxation be not essential, and venous oozing not objected to. Nitrous oxide mixed with 10 per cent. of oxygen is used for prolonged anæsthesia, the apparatus most commonly employed being Hewitt's. MacShail[5] gives two minutes as the period required to obtain unconsciousness with gas and oxygen. Lividity, stertor, and jactitation are absent, and the pupils are of normal size; the patient has no sense of discomfort whilst going under, and returns to consciousness in a few seconds. Muscular relaxation is, however, incomplete. After effects of unpleasant character are very rare. Fixation of the chest walls and spasm of the respiratory muscles is the greatest danger, but this method of anæsthetisation is undoubtedly the safest known. The chief objections to gas and oxygen are the cumbrous nature of the apparatus, and the difficulty of obtaining a uniform mixture of the gases, and of regulating their supply to the patient. Fifty gallons of nitrous oxide are required for every 15 minutes of anæsthesia. With this is mixed 5 to 10 per cent. of oxygen. Bellamy Gardner[6] speaks well of the use of the mixed gases for dental operations. Complete anæsthesia is marked by soft snoring, partial contraction of the pupils, convergence, downward movement, and vertical nystagmus of the eyeballs. For small surgical operations lasting from a quarter of an hour to 25 minutes, he found it necessary to introduce as much as 25 per cent. of oxygen in order to overcome rigidity, and even with this mixture reflex movements cannot be abolished, especially in operations on the rectum, urethra, &c. Another great drawback is that retching movements and sickness cannot be controlled by mere gas and oxygen, as with other anæsthetics. The mixture is totally unsuited for young children. The expense is great, and the apparatus unwieldy, two 50 gallon bottles of gas and one 25 gallon bottle of oxygen being required for half an hour's operation. A case of asphyxia during the administration of nitrous oxide and oxygen is recorded by Burny,[7] and cases of syncope and of spasmodic asthma from the same cause by Hilleard. None of these were fatal. Miller[8] gives the mortality from gas as 1 in 5,000,000, from ether 1 in 5,000 to 1 in 15,000, from chloroform 1 in 1,500. According to this authority it is a great advantage to administer gas and then ether; the comfort of the patient is greater, the time required to induce anæsthesia is lessened, the dangers of ether are minimised, and the after effects are less distressing. He uses for the ether a paper cylinder, six inches long, which has a pad of cotton wool fixed at one end on which the drug is poured. In this way the ether vapour is largely diluted with air, and asphyxial symptoms avoided.

**Ether.**—Carter[9] describes the Bennett gas and ether inhaler, largely used in America. He insists on the necessity of keeping the face piece and whole apparatus aseptic. The respiration only need be watched during ether administration. The breathing should be distinctly audible and slightly stertorous, and if either of these characters be lost, or if there be slight cough or swallowing movement, more ether is required. Deeply stertorous or embarrassed breathing, and a dilatation of the pupil not due to reflex cause, should lead to a diminution of the dose. The pupil should remain moderately contracted, and the pupil reflex should not respond to a lifting of the upper lid. From time to time the patient's head should be turned to one side, the neck extended upon the shoulders, and the chin lifted. This manœuvre raises the base of the tongue, and causes any mucus which may have accumulated to flow from the mouth. Gallant[10] recommends a modified Allis inhaler, which consists of a cone 8½ inches high placed over the patient's face, permitting a good admixture of air and a certain degree of warming. With regard to the after care, patients are allowed hot water *ad libitum* as soon as they are conscious. This lessens vomiting, quenches thirst, and flushes the bowels and kidneys. Milk and lime water should be the first nourishment given, and if abdominal distension is troublesome it may be relieved by massage of the colon or by high enemata. As soon as the dorsal decubitus becomes irksome turn the patient on the side.

Safe as ether generally is, there are yet from time to time cases recorded in which alarming symptoms of asphyxia arise. One such is recorded by McCardie in the *British Medical Journal* of January 20th, 1900, and a second by Shuter in the same journal of April 28th, 1900. Aldrich-Blake also records in the *Lancet*, January 6th, 1900, a death during ether administration. In this case there was, however, an abscess below the left ramus of the mandible and œdema glottidis—two conditions which should, as a rule, contra-indicate the use of ether unless there be heart weakness. Amongst recent new departures in ether inhalers we may mention Bennett's (see *Medical Record*, March 24th, 1900); Copestake's, used for ether, chloroform, or mixture; and Rose's, an especially light, compact instrument made by Meyer and Meltzer.

Recent work by Popoff[11] would seem to show that although transient albuminuria may follow the administration of ether, no permanent kidney mischief results. This is a matter of great importance, for the contrary view had been maintained by many observers, amongst whom were Thompson and Kemp.

[1] New York Med. Jour., Mar. 17, 1900. [2] Med. Rec., Mar. 10, 1900. [3] Lancet, Feb. 4, 1899. [4] New York Med. Jour., Aug. 5, 1900. [5] Caledon. Med. Jour., Oct., 1899. [6] Clinic. Jour., June 14, 1899. [7] Clinic. Jour., May 3, 1899. [8] Ann. of Surg., Dec., 1899. [9] Med. Rec., April 10, 1899. [10] Ibid., Dec. 30, 1899. [11] Ann. Gynæ. and Ped., April, 1899.

